# Promoting Mental Health and Preventing Mental Disorders: Adopting a Proactive Health Approach

**DOI:** 10.31083/AP39495

**Published:** 2025-04-03

**Authors:** Feng Jiang, Yi-lang Tang

**Affiliations:** ^1^School of International and Public Affairs, Shanghai Jiao Tong University, 200030 Shanghai, China; ^2^Institute of Healthy Yangtze River Delta, Shanghai Jiao Tong University, 200030 Shanghai, China; ^3^Institute of Health Policy, Shanghai Jiao Tong University, 200030 Shanghai, China; ^4^Department of Psychiatry and Behavioral Sciences, Emory University School of Medicine, Atlanta, GA 30329, USA; ^5^Mental Health Service Line, Atlanta VA Medical Center, Decatur, GA 30033, USA

Mental health is increasingly recognized as an integral component of overall well-being, a fact underscored by the World Health Organization’s declaration that “there is no health without mental health” [1]. The global burden of mental disorders is substantial, affecting individuals, families, and entire societies [2]. The chronic nature of many mental disorders makes it crucial to emphasize not only treatment but also the prevention and promotion of mental health. Preventive strategies can potentially reduce the onset and exacerbation of some mental disorders [3]. Mental disorders are influenced by a complex interplay of biological, psychological, and social factors. While biological and psychological determinants are often less amenable to direct control, many social factors, such as socioeconomic status, educational attainment, employment status, social support networks, and neighborhood environment, are relatively more modifiable [4]. Addressing these social determinants is key to enhancing overall mental well-being and preventing mental disorders.

To effectively tackle these challenges, shifting from a reactive model to a proactive health approach is necessary. Originating in China, the concept of proactive health integrates holistic medical principles and the disease prevention philosophy of Traditional Chinese Medicine, augmented with modern technology [5]. Traditional reactive mental health strategies primarily focus on symptom management through medication, therapy, and rehabilitation, but they can be costly, lead to delayed care, and are often stigmatized, limiting access. In contrast, proactive mental health approaches focus on fostering resilience and stress management skills, creating supportive environments, and prioritizing prevention and early intervention.

The essence of proactive health approach lies in the concept of initiative, characterized by self-initiated actions, a proactive mindset, and resilience in overcoming obstacles to achieve set goals. Unlike passive behavior, which is limited to following instructions, yielding to obstacles, and responding to environmental demands without a strategic plan, proactive health is grounded in a dynamic approach. It prioritizes health as a central objective in both individual and organizational agendas. It demands that health be regarded with high importance, encouraging individuals and institutions to actively pursue health-related goals by overcoming challenges through deliberate and sustained action. This shift towards proactive health represents a move from merely reacting to illness to a continuous, goal-oriented endeavor to maintain and enhance well-being. The initiative of the entire society and individuals in proactive health is the cornerstone of this approach. It also emphasizes the role of the community and individuals [6]. A proactive health framework emphasizes a comprehensive strategy for improving mental health outcomes by engaging all sectors of society [7]. This approach involves collaborations among global efforts, government, communities, medical institutions, and individuals to create a supportive environment that promotes mental well-being. Following the proactive health framework, we propose the following recommendations.

Firstly, global advocacy for mental health has recently gained significant momentum, particularly with the launch of various programs and initiatives. The global platforms have set forth key advocacy goals, including promoting mental health policy reform at both global and national levels, integrating mental health into universal health coverage (UHC), and ensuring the right to the highest attainable standard of mental health for all [8]. Achieving these goals requires the involvement of major international organizations, including mental health considerations across sectors and Sustainable Development Goals (SDGs), and addressing the social determinants of mental health to foster more supportive environments.

Secondly, the government plays a pivotal role in fostering a proactive health approach. Mental health must be prioritized within public health agendas, with regulations ensuring interdepartmental cooperation, and the optimization of community environments. Funding and support for preventive programs are crucial, and school-based initiatives should be a key focus. Evidence demonstrates that school programs effectively enhance mental health literacy, build resiliency, reduce stigma, and encourage help-seeking behaviors [9]. Investing in these programs can provide a strong foundation for lifelong mental health.

Thirdly, strengthening community engagement and fostering robust social support systems are vital for the success of proactive mental health initiatives. Community engagement involves actively involving local stakeholders, including parents, teachers, and community leaders, in the planning and execution of mental health programs. This collaborative approach ensures that the initiatives are tailored to meet the community’s specific needs and cultural contexts [10]. Additionally, building robust social support systems creates a network of care that can provide emotional, informational, and practical assistance to individuals.

Fourthly, medical institutions should take a proactive and integrated service model for mental disorders treatment and health promotion [11]. Healthcare institutions must assist the public in improving mental health literacy and personal initiative. Encouraging proactive management of mental disorders—such as through self-care practices, regular physical activity, and avoiding addictive substances such as tobacco or alcohol—is essential.

Fifthly, individuals should have a crucial role in cultivating healthy habits. Individuals should adopt healthy living practices tailored to their unique needs. In addition to the fundamental practices, incorporating stress management techniques into their daily routines can significantly benefit mental health. Staying informed about health-related knowledge and seeking regular professional advice can aid in the early detection and prevention of mental health issues. Proactive engagement in mental health care fosters a sense of personal responsibility and empowerment [12].

In summary, embracing this proactive health perspective represents a transformative approach to mental health promotion, as it allows us to move beyond traditional reactive approaches, focusing instead on cultivating strategies that promote long-term well-being and resilience. The path forward requires collaborative efforts, informed by evidence and guided by a commitment to holistic, preventive care. It’s important to recognize that proactive health strategies extend beyond mental health, encompassing the overall wellness of individuals. These strategies should be also integrated with existing health promotion and preventive medicine practices to ensure a holistic approach to health.
